# Trends in hospital admissions for adverse drug reactions in England: analysis of national hospital episode statistics 1998–2005

**DOI:** 10.1186/1472-6904-7-9

**Published:** 2007-09-25

**Authors:** Hitesh Patel, Derek Bell, Mariam Molokhia, Janakan Srishanmuganathan, Mitesh Patel, Josip Car, Azeem Majeed

**Affiliations:** 1 Charing Cross Hospital, London W6 8RF, UK; 2 Chelsea and Westminster Hospital, London SW10 9NH, UK; 3 Department of Epidemiology and Population Health, London School of Hygiene & Tropical Medicine, WC1E 7HT, UK; 4 St Mary's Hospital, London W2 1NY, UK; 5 Department of Primary Care and Social Medicine, Imperial College, London W6 8RF, UK

## Abstract

**Background:**

Adverse drug reactions (ADRs) are a frequent cause of mortality and morbidity to patients worldwide, with great associated costs to the healthcare providers including the NHS in England. We examined trends in hospital admissions associated with adverse drug reaction in English hospitals and the accuracy of national reporting.

**Methods:**

Data from the Hospital Episode Statistics database (collected by the Department of Health) was obtained and analysed for all English hospital episodes (1998–2005) using ICD-10 codes with a primary (codes including the words ('drug-induced' or 'due to') or secondary diagnosis of ADR (Y40–59). More detailed analysis was performed for the year 2004–2005

**Results:**

Between 1998 and 2005 there were 447 071 ADRs representing 0.50% of total hospital episodes and over this period the number of ADRs increased by 45%. All ADRs with an external code increased over this period. In 2005 the total number of episodes (all age groups) was 13,706,765 of which 76,692 (0.56%) were drug related. Systemic agents, which include anti-neoplastic drugs, were the most implicated class (15.7%), followed by analgesics (11.7%) and cardiovascular drugs (10.1%). There has been a 6 fold increase in nephropathy secondary to drugs and a 65% decline in drug induced extra-pyramidal side effects. 59% of cases involving adverse drug reactions involved patients above 60 years of age.

**Conclusion:**

ADRs have major public health and economic implications. Our data suggest that national Hospital Episode Statistics in England have recognised limitations and that consequently, admissions associated with adverse drug reactions continue to be under-recorded. External causes of ADR have increased at a greater rate than the increase in total hospital admissions. Improved and more detailed reporting combined with educational interventions to improve the recording of ADRs are needed to accurately monitor the morbidity caused by ADRs and to meaningfully evaluate national initiatives to reduce adverse drug reactions.

## Background

Adverse drug reactions cause considerable morbidity and mortality world-wide [1] and in many cases are avoidable. Pirmohamed and colleagues estimated that in England, adverse drug reactions were responsible for around 6.5% of all acute hospital admissions and at least 5,000 deaths per year [2]. In the USA, adverse drug reactions are one of the leading causes of death in the population [3]. Hence, adverse drug reactions have a major impact on public health, reducing patients' quality of life and imposing a considerable financial burden on the health care systems at a time when many health care systems are under considerable financial strain.

Drug reactions can be typically described in two groups. Type A reactions "intrinsic" (which are often dose-dependent) are relatively common. Type B reactions are usually more serious: idiosyncratic reactions that are not necessarily dose-dependent. We expect the majority of ADR admissions to be Type A reactions. Notification to pharmacovigilance agencies (e.g. the Medicines and Healthcare products Regulatory Agency yellow card in the UK) is designed to capture new ADRs not known at the marketing stage, i.e. usually but not exclusively, Type B reactions.

Adverse drug reactions are commonest amongst the elderly [2] This is not surprising as the elderly generally have the highest prevalence of long-term diseases [4]. Poly-pharmacotherapy, combined with a poorer physiological reserve, puts the elderly particularly at risk of adverse drug reactions [5]. Poly-pharmacotherapy amongst elderly patients is likely to increase still further through the implementation of 'pay for performance' schemes, such as the new contract for NHS general practitioners in the UK. This rewards tighter meeting of 'treatment targets' for specific long-term diseases, such as high blood pressure or high cholesterol, which commonly results in prescribing of higher doses of medication or poly-pharmacotherapy with higher number of different medications than in the past.

More evidence-based prescribing for long-term diseases will benefit patients, but may also increase the number of adverse drug reactions in the population merely because of the potential for increased drug reactions. The number of older people in the population of developed countries is also increasing rapidly. For example, in the United Kingdom, the number of people aged 65 years and over is predicted to increase by around 53% between 2001 and 2031 [6]. This increase in the elderly population combined with increased prescribing for long-term diseases is likely to result in an increase in the number of people at risk of suffering from adverse drug reactions.

Despite the importance of adverse drug reactions, methods for monitoring them are limited. Some adverse drug reactions are identified during clinical trials during drug development and testing, however, rare reactions may fail to be detected. Once a drug is marketed, detection of adverse drug reactions generally depends on notifications to regulatory authorities, such as the Medicines and Healthcare products Regulatory Agency, which runs the yellow card scheme in the United Kingdom. However, even for serious and well-recognized ADRs notification of adverse drug reactions from such spontaneous reporting schemes is low, often less than 10% and even lower where the association between the drug and the adverse event is unknown [7].

Hence, many adverse drug reactions do not become apparent until a drug has been in widespread use for several years. Consequently, current systems for the detection of ADRs have serious limitations. For example, the associations between COX-2 inhibitors and increased risk for myocardial infarction and stroke were only highlighted and reviewed after these drugs had been used for several years by hundreds of thousands of patients, although initial concerns were identified from trial data [8].

Other information on adverse drug reactions comes from ad-hoc studies. However, with a few exceptions, such as the study by Pirmohamed and colleagues in North-West England, [2] these previous studies have tended to be small (although with similar results; often based in one hospital) and many are now very old [1].

Hospital Episode Statistics (HES) are collected by the Department of Health and contain details of all admissions to NHS hospitals in England [9]. HES data includes private patients treated in NHS hospitals, patients who were resident outside of England and care delivered by treatment centres (including those in the independent sector) but funded by the NHS. HES were established in 1987 following a report on the collection and use of hospital activity information published by a steering group chaired by Dame Edith Körner [10]. Before 1987, only a 10 per cent sample of admitted patient records were collected nationally whereas HES collect a detailed record for each 'consultant episode' of admitted patient care delivered by NHS hospitals in England.

HES data are available for every financial year from 1989–90 onwards but the dataset has been modified over time to reflect changing administrative requirements and the introduction of new clinical classifications. For example, in 1995, the recording of diagnoses changed from the 9th to the 10th revision of the International Classification of Diseases (ICD) [9]. In the 2004–05 financial year, there were about 12.1 million hospital admissions, resulting in nearly 14 million consultant episodes, in England.

Waller and colleagues [11], studied HES data from 1996–2000, looking at 258, 222 episodes associated with confirmed ADRs and concluded that HES grossly underestimates the burden of ADRs. As part of preparatory work for projects on the epidemiology of adverse drug reactions, and educational interventions to improve recording of these reactions, we wished to examine the contribution of recorded adverse drug reactions to acute hospital admissions nationally. We aimed to examine the current epidemiology of hospital admission for adverse drug reactions, the age distribution of these admissions, and their impact on hospital activity. We also aimed to compare estimates of hospital admissions due to adverse drug reactions to those from previous studies to determine how well adverse drug reactions were being recorded in current practice and how much scope there was to improve recording.

## Methods

We obtained for the period 1998–2005 from the Department of Health, HES-records in which there was an ICD10 code containing the words 'drug-induced' or which indicated that a diagnosis was 'due to' a drug. We also studied codes in the range Y40 – Y59 ("correct drug properly administered in therapeutic or prophylactic dosage as the cause of any adverse effect"), otherwise known as 'external cause' (Table 1). This excludes accidental or intentional poisoning due to drugs. We studied codes that explicitly stated that the episode was caused by a drug. Thus, we did not include conditions like toxic epidermal necrolysis, which is invariably drug induced but does have other known causes.

**Table 1:** ICD-10 codes (Y40–Y59) for drugs, medicaments and biological substances causing adverse effects in therapeutic use

**ICD-10**	**External cause mortality/morbidity by:**
Y40	**Systemic antibiotics**: Penicillins, cefalosporins and other beta-lactam antibiotics, chloramphenicol, macrolides, tetracyclines, aminoglycosides, rifamycins, antifungals, others
Y41	**Other systemic anti-infectives and antiparasitics**: Sulphonamides, other anti-mycobacterial, anti-malarials, anti-protozoal, anti-helminthics, anti-virals
Y42	**Hormones and substitutes**: Glucocorticoids, thyroid hormones, anti-thyroids, insulin, oral hypoglycaemics, oral contraceptives, oestrogen and progestogen, anti-gonadotrophins, anti-oestrogens, anti-progestogens, androgens
Y43	**Systemic agents**: Anti-allergic and anti-emetic drugs, anti-neoplastic and immunosuppressive drugs, acidifying/alkalising agents
Y44	**Agents affecting blood constituents**: Iron preparations, anti-megaloblastic-anaemia preparations, anticoagulants, anticoagulant antagonists, antithrombotic drugs, thrombolytic drugs, blood products, plasma substitutes
Y45	**Analgesics, anti-pyretics and anti-inflammatory drugs**: Opioids and related analgesics, salicylates, propionic acid derivatives, nonsteroidal anti-inflammatory drugs, antirheumatics, 4-aminophenol derivatives
Y46	**Anti-epileptics and anti-parkinsonism drugs**: Succinimides, oxazolidinediones, hydantoin derivatives, deoxybarbiturates, iminostilbenes, valproic acid, anti-parkinsonism drugs, anti-spasticity drugs
Y47	**Sedatives, hypnotics and anti-anxiety drugs**: Barbiturates, benzodiazepines, cloral derivatives, paraldehyde, bromine compounds, sedative, hypnotic and antianxiety drug, unspecified
Y48	**Anaesthetics and therapeutic gases**: Inhaled/parenteral anaesthetics, local anaesthetics, therapeutic gases
Y49	**Psychotropic drugs**: Tricyclic and tetracyclic antidepressants, monoamine-oxidase-inhibitor, phenothiazine antipsychotics and neuroleptics, butyrophenone and thioxanthene neuroleptics, other antidepressants, antipsychotics and neuroleptics
Y50	**Central nervous system stimulants**: Analeptics, opioid receptor antagonists, methylxanthines, other central nervous system stimulants
Y51	**Drugs primarily affecting the autonomic nervous system**: Anticholinesterase agents, cholinergics, ganglionic blocking drugs, anticholinergics, antimuscarinics, spasmolytics, alpha-adrenoreceptor agonists/antagonists, beta-adrenoreceptor agonists/antagonists, centrally acting and adrenergic-neuron-blocking agents
Y52	**Agents affecting the cardiovascular system**: Cardiac-stimulant glycosides, calcium-channel blockers, other anti-dysrhythmic drugs, other coronary vasodilators, angiotensin-converting-enzyme inhibitors, other anti-hypertensives, anti-hyperlipidaemic and anti-arteriosclerotic drugs, peripheral vasodilators, anti-varicose drugs
Y53	**Agents affecting the gastrointestinal system**: Antacids, anti-gastric-secretion drugs, laxatives, anti-diarrhoeal, emetics
Y54	**Agents affecting water-balance and mineral and uric acid metabolism**: Mineralocorticoids, mineralocorticoid antagonists, carbonic-anhydrase inhibitors, benzothiadiazine derivatives, other diuretics, electrolytic, caloric and water-balance agents, agents affecting calcification, agents affecting uric acid metabolism
Y55	**Agents acting on smooth and skeletal muscles and the respiratory system**: Oxytocic drugs, skeletal muscle relaxants, anti-tussives, expectorants, anti-common-cold drugs, anti-asthmatics
Y56	**Topical agents primarily affecting skin and mucous membrane**: Local anti-fungal, anti-infective, anti-inflammatory drugs, anti-pruritics, local detergents, emollients, keratolytics, ophthalmological drugs, otorhinolaryngological drugs, dental drugs
Y57	**Other and unspecified drugs**: Appetite depressants, lipotropic drugs, antidotes and chelating agents, alcohol deterrents, x-ray contrast media, vitamins
Y58	**Bacterial vaccines**
Y59	**Other vaccines**: Viral/rickettsial/protozoal vaccines, immunoglobulin

Includes: Correct drug properly administered in therapeutic or prophylactic dosage as the cause of any adverse effect Excludes: Accidental overdose of drug or wrong drug given or taken in error (X40–X44); accidents in the technique of administration of drugs, medicaments and biological substances in medical and surgical procedures (Y60–Y69)

HES records contain a main diagnosis field and up to 13 secondary diagnosis fields (seven before 2002/3). We examined the number of episodes per year and for the most recent year for which data was available (2004/05); we also examined several other measures, including the number of admissions by age and the total bed days used by patients with diagnoses of adverse drug reactions. An episode is defined as the time a patient spends under the care of one consultant. Most admissions result in just one episode of care and hence episodes are a reasonably good proxy for admissions.

HES data is collected in financial year from 1 April to 31 March the following year. Our data is presented in this format. HES data is collected comprehensively and undergoes extensive processing and validation to maintain quality. This process is detailed on their official website [9]. Some of the data presented changed classification during the time period and it became dubious whether the episode was related exclusively to an ADR. These data were not included and are marked as U/C (unclassified) in the tables.

## Results

### Overall burden of hospital admissions for ADRs

Table 2 summarises the total number of episodes and the total number of episodes associated with ADRs. In our seven year study period, there were 88,822,005 total hospital episodes and 447,041 episodes with a diagnostic code indicative of ADRs (0.5%). Of these 68,971 (0.08%) were primary diagnoses and 378,070 (0.4%) were of 'external cause'.

**Table 2:** Total number of Hospital Episode Statsitics for which there was a primary diagnosis or 'external cause' of ADR 1998–2005

	**1998–9**	**1999–00**	**2000–1**	**2001–2**	**2002–3**	**2003–4**	**2004–5**	**% Change 1998–2005**
**Total No. of Episodes**	11,983,893	12,167,574	12,674,277	12,357,360	12,757,656	13,174,480	13,706,765	14.4
**No. with 'drug induced' codes**	10506	10105	9823	9442	9246	9453	10396	-1.0
**No. with external cause codes**	42,555	47,385	50,113	52,160	56,943	62,618	66,296	55.8
**Total Adverse Drug Reactions**	53,061	57,490	59,936	61,602	66,189	72,071	76,692	44.5

### Change in burden

Between 1998–2005, the total number of hospital episodes increased by 14% but the total number of reported episodes linked with ADRs increased by 45% (largely due to an increase in external cause codes). Table 3 and 4 highlight the burden of ADRs according to ICD-10 code. Drug induced haemolytic anaemia (927%), nephropathy (590%), adrenocortical failure (146%), cardiomyopathy (144%) and aplastic anaemia (139%) were the five fastest growing ADRs as a primary diagnosis (Table 3). In contrast, the top five fastest growing ADRs as external causes were drugs relating to: water balance (164%), autonomic system (108%), central nervous system stimulants (102%), cardiovascular system (92%) and biologicals/vaccines (77%). Primary diagnosis of ADRs decreased by 1% during this study. Reported rates under the categories of 'drug-induced extra-pyramidal side effects' (-65%) and 'malignant hyperpyrexia' (-33%) showed the largest declines (Table 3).

**Table 3:** Annual number of Hospital Episode Statistics finished consultant episodes with a primary diagnosis for an ADR (ICD-10 code)

**ICD-10**		**1998–9**	**1999–00**	**2000–1**	**2001–2**	**2002–3**	**2003–4**	**2004–5**	**% Change 1998–2005**
**D59.0/2**	**Drug induced haemolytic anaemia**	15	30	28	37	34	36	154	926.7
**D61.1**	**Drug induced aplastic anaemia**	101	116	114	186	182	162	241	138.6
**E03.2**	**Hypothyroidism due to medicaments**	23	23	12	12	19	13	26	13.0
**E27.3**	**Drug induced adrenocortical failure**	24	22	24	38	39	53	59	145.8
**F11**	**Mental disorders due to opioids**	4360	4287	4398	4187	3916	3690	3746	-14.1
**F13**	**Mental disorders due to sedatives/hypnotics**	223	247	247	239	232	206	189	-15.2
**F19**	**Mental disorders due to multiple psychoactive drugs**	3513	3137	2725	2643	2601	2690	2775	-21.0
**G21.0**	**Malignant neuroleptic syndrome**	101	106	121	108	102	93	128	26.7
**G21.1**	**Drug induced Parkinsonism**	145	115	124	133	112	108	149	2.8
**G24.0**	**Drug induced dystonia**	149	130	145	130	115	152	109	-26.8
**G25.1/4/6**	**Drug induced extrapyramidal syndrome/chorea/tics**	136	146	110	125	44	51	48	-64.7
**G72.0**	**Drug induced myopathy**	47	53	51	51	57	39	55	17.0
**H91.0**	**Ototoxic hearing loss**	2	5	3	3	2	U/C^1^	U/C	U/C
**I42.7**	**Drug induced cardiomyopathy**	25	25	53	38	67	77	61	144.0
**J70.2/3/4**	**Drug induced interstitial lung disorders**	54	45	43	43	86	71	94	74.1
**K71**	**Drug induced liver disease**	406	318	372	351	459	437	448	10.3
**L56.0/1**	**Drug induced phototoxicity**	4	5	2	4	1	3	5	25.0
**M10.2**	**Drug induced gout**	30	27	13	24	22	22	34	13.3
**M32.0**	**Drug induced systemic lupus erythematosus**	17	20	12	13	14	10	26	52.9
**M34.2**	**Drug induced systemic sclerosis**	3	3	4	3	U/C	U/C	U/C	U/C
**N14**	**Drug induced nephropathy**	60	69	92	85	100	318	414	590.0
**T88.3**	**Malignant hyperthermia due to anaesthesia**	6	4	10	3	3	5	4	-33.3
**T88.6**	**Drug induced anaphylaxis**	366	435	399	384	375	469	563	53.8
**T88.7**	**Unspecified adverse drug effect**	696	737	721	602	664	748	1068	53.4

**Total**		**10506**	**10105**	**9823**	**9442**	**9246**	**9453**	**10396**	**-1.0**

^1^U/C – unclassified: data not available due to a coding change

**Table 4:** Annual number of HES finished consultant episodes with an 'external code' for an adverse drug reaction

**ICD-10**		**1998–9**	**1999–00**	**2000–1**	**2001–2**	**2002–3**	**2003–4**	**2004–5**	**% Change 1998–2005**
**Y40**	**Systemic antibiotics**	4,206	4,212	4,533	4,341	4,697	5,624	6,449	53.3
**Y41**	**Other systemic anti-infectives/anti-parasitics**	829	816	1,017	945	1,195	1,134	1,453	75.3
**Y42**	**Hormones (including synthetic, antagonists)**	4,547	5,088	4,934	5,113	5,803	5,461	5,319	17
**Y43**	**Primarily systemic agents**	7,501	8,271	9,078	9,877	10,766	11,226	12,054	60.7
**Y44**	**Agents primarily affecting blood constituents**	4,062	4,483	4,723	4,797	5,230	5,995	4,272	5.2
**Y45**	**Analgesics/antipyretics/anti-inflammatory**	5,951	6,726	6,787	6,819	7,540	8,079	9,004	51.3
**Y46**	**Antiepileptics/antiParkinsonism drugs**	1,230	1,267	1,224	1,340	1,401	1,501	1,628	32.4
**Y47**	**Sedatives, hypnotics, anti-anxiety drugs**	370	400	450	430	480	586	560	51.4
**Y48**	**Anaesthetics, therapeutic gases**	414	538	502	440	505	521	531	28.3
**Y49**	**Psychotropic drugs**	1,653	1,678	2,008	1,992	1,953	2,364	2,544	53.9
**Y50**	**Central nervous system stimulants**	46	67	75	76	69	71	93	102.2
**Y51**	**Drugs affecting autonomic nervous system**	1,702	2,056	2,277	2,470	2,686	3,239	3,532	107.5
**Y52**	**Agents primarily affecting cardiovascular system**	4,044	4,752	5,376	5,667	6,234	6,836	7,768	92.1
**Y53**	**Agents primarily affecting gastrointestinal system**	398	410	486	416	461	579	656	64.8
**Y54**	**Agents affecting water/mineral balance/uric acid**	2,136	2,617	2,945	3,526	4,151	5,118	5,638	164
**Y55**	**Agents affecting muscle/respiratory system**	312	299	342	321	323	430	422	35.3
**Y56**	**Topical agents affecting skin/ENT, dental**	1,138	1,236	983	1,202	1,065	1,079	1,224	7.6
**Y57**	**Other and unspecified medicaments**	1,524	1,715	1,780	1,907	1,906	2,014	2,398	57.3
**Y58**	**Bacterial vaccines**	226	347	255	182	197	356	281	24.3
**Y59**	**Other vaccines/biologicals**	266	407	338	299	281	405	470	76.7

**Total**		42,555	47,385	50,113	52,160	56,943	62,618	66,296	**55.8**

### Detailed overview for 2004–5

In 2004, there were 10,396 (0.08%) ADR admissions associated with a primary diagnosis and 66,296 (0.5%) ADRs as a secondary cause. Combined, they account for 0.56% of hospital episodes. The three commonest classes of adverse drug reaction, classified as an external cause (Table 4) resulting in hospital admission were systemic agents (Y43); analgesics, antipyretics and anti-inflammatory drugs (Y45); and systemic antibiotics (Y40). 'Mental disorders' secondary to opioids and psychoactive drugs reflected 63% of all the primary diagnoses of ADR (Table 3).

Most hospital episodes associated with adverse drug reactions occurred in the elderly. In 2004–5, 59% of hospital episodes in which there was an external ICD10 code for an adverse drug reaction occurred in people aged 60 years and over. The mean age of ADRs was 60 (Table 5, 6). Younger patients tended to have ADRs from vaccines and psychoactive drugs.

**Table 5:** Number of HES finished consultant episodes with a primary diagnosis of an ADR in 2004/05: mean age of admissions, age & gender distribution of episodes and total bed days

**ICD-10**		**Male %**	**Mean age**	**Age 0–14 (%)**	**Age 15–59 (%)**	**Age 60–74 (%)**	**Age 75+ (%)**	**Total Bed days**
**D59.0/2**	**Drug induced haemolytic anaemia**	49	63	0 (0.0)	52 (0.5)	75 (0.8)	27 (0.3)	160
**D61.1**	**Drug induced aplastic anaemia**	49	64	2 (0.0)	89 (0.9)	74 (0.7)	73 (0.7)	604
**E03.2**	**Hypothyroidism due to medicaments**	42	72	0 (0.0)	6 (0.1)	3 (0.0)	17 (0.2)	191
**E27.3**	**Drug induced adrenocortical failure**	41	48	11 (0.1)	22 (0.2)	12 (0.1)	14 (0.1)	584
**F11**	**Mental disorders due to opioids**	66	32	14 (0.1)	3681 (36.9)	33 (0.3)	14 (0.1)	47390
**F13**	**Mental disorders due to sedatives/hypnotics**	54	47	6 (0.1)	123 (1.2)	33 (0.3)	27 (0.3)	4922
**F19**	**Mental disorders due to multiple psychoactive drugs**	75	31	14 (0.1)	2734 (27.4)	17 (0.2)	10 (0.1)	64152
**G21.0**	**Malignant neuroleptic syndrome**	67	50	0 (0.0)	88 (0.9)	28 (0.3)	12 (0.1)	2,315
**G21.1**	**Drug induced Parkinsonism**	43	77	1 0.0)	11 (0.1)	44 (0.4)	93 (0.9)	1,964
**G24.0**	**Drug induced dystonia**	47	43	10 (0.1)	65 (0.7)	16 (0.2)	18 (0.2)	550
**G25.1/4/6**	**Drug induced extrapyramidal syndrome/chorea/tics**	40	64	2 (0.0)	15 (0.2)	18 (0.2)	13 (0.1)	407
**G72.0**	**Drug induced myopathy**	51	65	1 (0.0)	12 (0.1)	20 (0.2)	22 (0.2)	517
**H91.0**	**Ototoxic hearing loss**	U/C	U/C	U/C	U/C	U/C	U/C	U/C
**I42.7**	**Drug induced cardiomyopathy**	48	43	7 (0.1)	42 (0.4)	7 (0.1)	5 (0.1)	376
**J70.2/3/4**	**Drug induced interstitial lung disorders**	49	64	3 (0.0)	24 (0.2)	40 (0.4)	27 (0.3)	858
**K71**	**Drug induced liver disease**	43	47	12 (0.1)	315 (3.2)	50 (0.5)	71 (0.7)	3408
**L56.0/1**	**Drug induced phototoxicity**	0	47	1 (0.0)	2 (0.0)	2 (0.0)	0 (0.0)	17
**M10.2**	**Drug induced gout**	53	75	0 (0.0)	4 (0.0)	9 (0.1)	21 (0.2)	237
**M32.0**	**Drug induced systemic lupus erythematosus**	27	43	0 (0.0)	19 (0.2)	2 (0.0)	5 (0.1)	69
**M34.2**	**Drug induced systemic sclerosis**	U/C	U/C	U/C	U/C	U/C	U/C	U/C
**N14**	**Drug induced nephropathy**	2	58	0 (0.0)	3 (0.0)	5 (0.1)	3 (0.0)	111
**T88.3**	**Malignant hyperthermia due to anaesthesia**	75	20	2 (0.0)	2 (0.0)	0 (0.0)	0 (0.0)	8
**T88.6**	**Drug induced anaphylaxis**	37	52	19 (0.2)	326 (3.3)	130 (1.3)	87 (0.9)	883
**T88.7**	**Unspecified adverse drug effect**	33	50	80 (0.8)	565 (5.7)	203 (2.0)	217 (2.2)	2,471

**Total**		33	40	185 (1.9)	8200 (82.1)	821 (8.2)	776 (7.8)	132194

**Table 6:** Number of HES finished consultant episodes with an external ICD10 cause for an adverse drug reaction in 2004/05: mean age of admissions, age & gender distribution of episodes and total bed days

**ICD-10**		**Male %**	**Mean age**	**Age 0–14 (%)**	**Age 15–59 (%)**	**Age 60–74 (%)**	**Age 75+ (%)**	**Total bed days**
**Y40**	**Antibiotics**	40	57	467 (0.7)	2468 (3.7)	1433 (2.2)	2064 (3.1)	48,868
**Y41**	**Other anti-infectives**	39	55	48 (0.1)	776 (1.2)	245 (0.4)	362 (0.5)	9,280
**Y42**	**Hormones**	37	61	173 (0.3)	2051 (3.1)	1571 (2.4)	1513 (2.3)	31,323
**Y43**	**Systemic agents**	46	52	1475 (2.2)	5123 (7.7)	4107 (6.2)	1323 (2.0)	83,049
**Y44**	**Blood constituents**	47	70	37 (0.1)	830 (1.3)	1331 (2.0)	2072 (3.1)	31,919
**Y45**	**Analgesics, antipyretics, anti-inflammatory**	44	66	101 (0.2)	2756 (4.2)	2392 (3.6)	3746 (5.7)	50,118
**Y46**	**Antiepileptics, antiparkinsonism**	51	59	69 (0.1)	641 (1.0)	434 (0.7)	484 (0.7)	15,002
**Y47**	**Sedatives, hypnotics, anti-anxiety**	43	56	54 (0.1)	210 (0.3)	104 (0.2)	190 (0.3)	4,782
**Y48**	**Anaesthetics, therapeutic gases**	47	50	32 (0.0)	275 (0.4)	145 (0.2)	78 (0.1)	2,263
**Y49**	**Psychotropic drugs**	39	65	33 (0.0)	822 (1.2)	610 (0.9)	1076 (1.6)	25,733
**Y50**	**Central nervous system stimulants**	46	51	6 (0.0)	47 (0.1)	17 (0.0)	23 (0.0)	404
**Y51**	**Autonomic nervous system**	49	73	16 (0.0)	473 (0.7)	1037 (1.6)	2006 (3.0)	16,886
**Y52**	**Cardiovascular system**	47	75	15 (0.0)	870 (1.3)	2210 (3.3)	4669 (7.1)	48,189
**Y53**	**Gastrointestinal system**	39	62	30 (0.0)	222 (0.3)	163 (0.2)	239 (0.4)	3,597
**Y54**	**Water-balance, mineral and uric acid**	33	77	13 (0.0)	411 (0.6)	1360 (2.1)	3848 (5.8)	42,568
**Y55**	**Smooth/skeletal muscle, respiratory system**	39	56	24 (0.0)	182 (0.3)	105 (0.2)	111 (0.2)	2,232
**Y56**	**Topical agents**	42	58	68 (0.1)	459 (0.7)	344 (0.5)	351 (0.5)	5,779
**Y57**	**Other unspecified drugs**	43	60	87 (0.1)	894 (1.4)	649 (1.0)	760 (1.1)	15,835
**Y58**	**Bacterial vaccines**	52	10	243 (0.4)	15 (0.0)	14 (0.0)	9 (0.0)	296
**Y59**	**Other vaccines, biological substance**	51	28	211 (0.3)	162 (0.2)	57 (0.1)	40 (0.1)	1,562

**Total**		**43**	**63**	**3202 (4.8)**	**19687 (29.7)**	**18328 (27.7)**	**24964 (37.7)**	**439,685**

**Combined Primary Diagnosis and External Cause**		**42**	**60**	**3387 (4.4)**	**27887 (36.6)**	**19149 (25.1)**	**25740 (33.8)**	**571,879**

The majority of adverse drug reactions were reported in females (58%). By contrast, females accounted for 51% of all hospital admissions in 2004–05. Men were more likely to suffer from mental disorders due to psychoactive drugs and women were more likely to suffer from drug induced nephropathy and systemic lupus erythematosus. On average, each episode associated with an adverse drug reaction required a hospital admission lasting 9.7 days (compared with 7.1 days as the mean length of stay for admissions due to any cause). Episodes with a record of an adverse drug reaction accounted for 0.8% (439,685/54,554,697) of total bed days in 2004–05.

## Discussion

This national study on time trends for hospital admissions for adverse drug reactions in England is unique in covering such a large population and for such long time period. Our data show that the number of admissions linked to adverse drug reactions has increased substantially over the time period covered by the study.

The reported increase of ADRs by 45% may be accounted by improving record keeping due to increased awareness, a general increase in ADRs against a background of an increasingly elderly population, the introduction of new drugs and poly-pharmacotherapy because of increased pressure to prescribe medication for chronic diseases. For example, Swedish studies have shown a substantial increase over the last 30 years in the average number of drugs prescribed to patients who were admitted to hospital because of an ADR [12,13]. Our data shows a continuation of the trends in Waller's and colleague's paper, which reported a 40% increase in ADRs between 1996–2000. When interpreting any results, we must bear in mind the limitations of this study, which are outlined below. However, even in 2004/05, adverse drug reactions accounted for only about 0.56% of all emergency hospital episodes in that year. This is substantially less than the 5% of emergency hospital admissions generally quoted from other studies, suggesting there is considerable under-recording of adverse drug reactions in routine hospital activity data.

The strengths of our study over earlier work include its longitudinal nature and its completeness in that it covers all NHS hospital admissions in England. All submitted HES data are verified, validated and where appropriate overwritten to maintain accuracy. Furthermore, the data are compared with the independent Körner aggregate returns (KP 70) to ensure that all consultant episodes are captured [14]. However, our data has the weaknesses associated with routinely collected data, such as missing, incomplete or inaccurate data. It is likely that the effects of these would lead us to under-estimate the true burden of ADRs on the NHS. HES data are also collected locally at hospitals in a process that involves different coders and clinicians, and this may introduce variability in coding practice between hospitals. Consequently, there are errors in HES data with, for example, up to 22% error rates in codes reported in plastic surgery [15]. Despite this, HES remain a key source of information on hospital activity in England's NHS. We did not have access to mortality data associated with our hospital episodes, which would have given more insight into the severity of ADRs associated with hospital admissions.

### Potential sources of bias

We do not have data on the total number of each drug group prescribed and hence we are unable to conclude whether adverse drug reactions were more common with certain drug groups because they are prescribed more often or whether the drugs are more likely to lead to an adverse event. The number of admissions is likely to reflect a combination of these two factors. For example, the increase in admissions due to agents affecting the cardiovascular system (Y52) may reflect increased use of these drugs to treat conditions such as hypertension or heart failure in the general population.

The number of medications a patient takes is associated with the risk of an adverse drug reaction, with the mean rate increasing by 10% with each extra medication prescribed [16]. HES data does not allow us to monitor the effects of poly-pharmacotherapy on adverse drug reaction trends directly. This could be done by using data from computerised primary care records held by general practitioners.

We did not have access to the raw data and thus were not able to identify duplicates, which is another limitation of HES data. However, the purpose of this study was to see whether the freely available data (without further data manipulations) from HES has a role in monitoring ADRs and evaluating interventions to reduced ADRs.

### Comparison with other studies

#### Overall trends

There are limited recent studies examining the epidemiology of adverse drug reactions, especially in England [1]. Pirmohamed et al undertook a 6 month prospective study of admissions to two North England hospitals. However, even their study, which is widely cited and regarded as one of the best sources of information on the epidemiology of adverse drug reactions, reported on only 1225 admissions linked to adverse drug reactions. Their estimate of that 6.5% of emergency admissions were associated with adverse drug reactions may be an over-estimate because they excluded groups such as children, women presenting with obstetrics and gynaecological problems; both women of child-bearing age and women are likely to have relatively few admissions due to adverse drug reactions. A recent two year survey of the National Electronic Surveillance – All Injury Programme (NEISS-AIP) consisting of 63 US hospitals that are nationally representative, which analysed over 21 000 emergency department visits due to adverse drug reactions over two years, estimated they accounted for 0.6% of all emergency department visits [17].

Our estimates of ADRs from HES suggest an under-reporting in concordance with the conclusions of Waller and colleagues [11]. Our data showed a higher incidence of ADRs than Waller's study, which is difficult to explain. It may represent an increasing trend in ADRs, better record keeping, or improved vigilance. The present study does show emerging trends of drug induced nephropathy and reduced incidence of side effects from Parkinson's disease. We could theorise reasons for this but it would be difficult to interpret without knowing the specific medicines causing these trends.

In the two most recent studies, length of stay in patients with adverse drug reactions was 8 (median) [2]and 10.6 (mean) [3] days and is longer than current average hospital stays. Our study gives a similar mean value. The cost of a hospital bed in the UK is €228 per day [18], however, it is difficult to estimate the exact cost associated with hospital episode related adverse drug reactions, given the degree of underreporting. Shorter hospital stays may lead to underreporting of adverse drug reactions.

#### Age

The elderly are known to experience more type A drug reactions (predictable reactions related to the pharmacodynamics of the drug) [5]. The NEISS-AIP paper found that individuals over 65 years of age accounted for 50% of all hospitalisations for an adverse drug reactions [12], which is comparable to our figure of 65% for people over 60 (21% of the UK population).

The largest burden of ADRs in the 0–14 year age group (44%) was with systemic agents. Approximately 42% of paediatric prescriptions are 'off-label' (drug use outside its licence) and these are more likely to cause ADRs [19]. It should be possible to examine if the availability of new prescribing guidelines following the publication of the specific British National Formulary for Children in 2005 has had an effect on ADRs and off label drug use in this cohort.

#### Sex

Pirmohamed et al. reported that 59% the patients admitted with an adverse drug reaction were female. Our data provides further support to this. Further research to elucidate whether this relationship occurs because women are more likely to develop adverse drug reactions or because women are more likely to report adverse drug reactions is needed. It is widely known that women tend to present more to their doctors for treatment and consequently would be expected to experience more ADRs, however, our data showed that for non-ADR causes women had a similar number of hospital episodes as men. Rademaker has suggested that pharmacological, immunological and hormonal differences and the fact that women take more medications may explain some gender differences [20].

#### Major drug groups

Systemic agents (Y43), which includes cancer chemotherapy, were the most common cause of admissions in our study, similar to the findings of Waller and colleagues [11]. This will require further attention as estimates of the life time risk of cancer are reported to be 1 in 3 [21]. Increase in ADRs may also be due to more aggressive management made permissible by bone marrow rescue with growth colony stimulating factors (neutropenia is a common serious side effect of chemotherapy). The only other comparable study to ours in terms of size and generalisability is the US NEISS-AIP [13]. Even though their therapeutic drug categories are different to ours, their top five classes included (in brackets are approximately equivalent ICD-10 codes): central nervous system agents (Y45–50), antimicrobial agents (Y40–1), hormone modifying agents (Y42), haematological and oncology agents (Y43–4) and cardiovascular agents (Y52), which is broadly similar to findings using HES data for England.

### Implications for policy & conclusions

Adverse drug reactions have major clinical, public health and economic implications but our data suggest that admissions associated with adverse drug reactions are not being well recorded by NHS hospitals in England. A key requirement would be to include more detailed breakdown of drugs implicated in ADRs. Several European countries also include causality assessment criteria by which likelihood of ADR being related to a particular drug can be estimated. Other suggestions include linking these episodes to prescribing in primary care or pharmacies.

The current deficiencies in routine systems for monitoring ADRs have several important implications. Firstly, the NHS is not able to monitor accurately the burden of ill-health and mortality, or the financial costs, of adverse drug reactions. Secondly, potentially valuable information that could help us understand the aetiology of adverse drug reactions and identify patients for further studies (for example pharmacogenetic studies), is not being recorded. Finally, if accurate information on admissions due to adverse drug reactions is not available, then the NHS can not assess the impact of interventions to improve prescribing. There is strong support for such interventions but if they are introduced, without accurate routine information on the burden of adverse drug reactions, we have no way of measuring their clinical and their cost effectiveness.

Locally, we are developing educational interventions and mechanisms to feedback information on rates of correct medical prescribing, drug interactions and of recording of adverse drug reactions to hospital doctors and clinical coders in hospitals linked to Imperial College's School of Medicine. We will be evaluating the impact of such interventions on the recording of adverse drug reactions and using the information generated to develop and monitor the impact of preventive strategies to prevent such events. Hospitals in other areas should also consider adopting their own interventions to improve the identification and coding of admissions linked to adverse drug reactions.

## Competing interests

The author(s) declare that they have no competing interests.

## Authors' contributions

AM, DB, JC conceived the study, acted as supervisors and made critical contributions to the manuscript at each stage. HP, MP and JS obtained and analysed the data, and prepared the initial drafts. MM contributed significantly to revisions.

## Pre-publication history

The pre-publication history for this paper can be accessed here:


